# Genotype–Phenotype Correlations and Shifting Diagnosis Age in Turkish Mucopolysaccharidosis Type II Patients: A Multicenter Retrospective Study

**DOI:** 10.3390/diagnostics15212773

**Published:** 2025-10-31

**Authors:** Havva Yazıcı, Esra Kara, Fatma Derya Bulut, Merve Yoldaş Çelik, Burcu Köşeci, Fehime Erdem, Ezgi Burgaç, Ayşe Yüksel Yanbolu, İrem Kaplan, Asude Durmaz, Ayça Aykut, Ebru Canda, Deniz Kor, Sema Kalkan Uçar, Eser Sözmen, Mahmut Çoker, Halise Neslihan Önenli Mungan

**Affiliations:** 1 Department of Pediatrics, Division of Pediatric Metabolism and Nutrition, Ege University Faculty of Medicine, Izmir 35040, Turkey; drmerveyoldas@yahoo.com (M.Y.Ç.); fehimeerdem@gmail.com (F.E.); ayseyuksel81@hotmail.com (A.Y.Y.); ebru.canda@ege.edu.tr (E.C.); sema.kalkan.ucar@ege.edu.tr (S.K.U.); mahmut.coker@ege.edu.tr (M.Ç.); 2 Department of Pediatrics, Division of Pediatric Metabolism and Nutrition, Çukurova University Faculty of Medicine, Adana 01330, Turkey; esraulas8280@gmail.com (E.K.); deryaozduran@yahoo.com (F.D.B.); drburcukoseci@gmail.com (B.K.); ezgi_irmak@yahoo.com (E.B.); iremkekec@hotmail.com (İ.K.); dozonur@yahoo.com (D.K.); munganhno@gmail.com (H.N.Ö.M.); 3 Department of Medical Genetics, Ege University Faculty of Medicine, Izmir 35040, Turkey; asudealpman@gmail.com (A.D.); ayca.aykut@ege.edu.tr (A.A.); 4 Department of Biochemistry, Ege University Faculty of Medicine, Izmir 35040, Turkey; esersoz@yahoo.com

**Keywords:** genotype, Hunter syndrome, *IDS*, idursulfase, mucopolysaccharidosis type II

## Abstract

**Background/Objectives**: Mucopolysaccharidosis type II (MPS II) is an inherited metabolic disorder characterized by progressive neurologic and extra-neurologic findings. We aimed to explore the age at symptom onset and at diagnosis as well as contribute to the phenotype–genotype association with new observations of MPS II based on a broad series of patients in Turkey. **Methods**: The presented study was retrospective and descriptive. Data on molecular analysis results, the age of onset and diagnosis, diagnostic delays, neurologic and extra-neurologic symptoms, enzyme and urine glycosaminoglycan (GAG) level results, brain magnetic resonance imaging, echocardiography, and electromyography were reviewed. **Results:** A total of 46 MPS II patients from 40 families were involved. The mean diagnosis age was 40.1 ± 46.8 months, and the diagnostic delay was 19.7 ± 40.4 months. While the mean age of diagnosis of the first cases in the families was 45 ± 24 months, the mean age of diagnosis of the second cases was 14 ± 15 months. The mean age at diagnosis was 42 ± 18 months in the patient group born before 2010, while it was 28 ± 22 months in the patient group born in 2010 and after. The last measurement of the height SDS value showed a significant difference (*p* = 0.004) between the groups that started ERT before the age of three and those who began ERT at the age of three and above. Five patients showed an attenuated phenotype without neurologic involvement. The sequencing of the *IDS* gene revealed 25 distinct variants, with 8 novel variants that have yet to be documented in the existing literature. **Conclusions**: The findings from the observations of this Turkish MPS II cohort emphasize that the actual prevalence of MPS II is probably underestimated and that it has a broad spectrum of clinical phenotypes, even without neurological impairment. In children, specific warning signs—including a coarse facial appearance, abdominal distension, speech delays, and macrocephaly—should raise suspicion and prevent delays in diagnosis. Conducting urine GAG and enzyme analyses is crucial for cases with clinical suspicion. Our data showed that the age of diagnosis tended to decrease over the years.

## 1. Introduction

Mucopolysaccharidosis type II (OMIM#309900) (MPS II), or Hunter syndrome, is a rare lysosomal storage disorder. It is the only X-linked recessive inherited mucopolysaccharidosis type. The disorder arises from the lack of the iduronate 2-sulfatase (I2S) enzyme encoded by the *IDS* (iduronate-2-sulfatase) gene (OMIM*300823) [1].

I2S is one of the enzymes involved in the breaking down of glycosaminoglycans (GAGs), especially heparan sulfate (HS) and dermatan sulfate (DS), in lysosomes. Partially degraded HS and DS accumulate and lead to multisystemic changes, including visceromegaly, a short stature, dysostosis multiplex, an inguinal hernia, a coarse face, hearing loss, respiratory problems, heart valve insufficiency, cardiomyopathy, and occasionally, impacts on the nervous system. However, corneal clouding, a consistent quality of other mucopolysaccharidoses, is rare in MPS II.

MPS II is generally categorized into two clinical subtypes: severe (neuronopathic) and attenuated (non-neuronopathic) forms. The most frequent form is the severe form, which is characterized by an early onset of symptoms between 12 and 36 months and progressive neurologic system impairment. Patients with the attenuated form are distinguished by normal intelligence.

The recommended standard screening test for mucopolysaccharidosis type II (MPS II) is the analysis of urinary GAGs. The second step on the road to diagnosis is measuring I2S enzyme activity. The final step for the diagnosis is performing a molecular analysis of the *IDS* gene. More than 600 disease-causing pathogenic variants have been identified in the *IDS* gene, most of which are missense. The majority of MPS II patients are males due to the X-linked inheritance of this condition. However, females can also be affected due to chromosomal translocations, X chromosome monosomy, or skewed X chromosome inactivation [2].

Enzyme replacement therapy (ERT) (idursulfase) has been available for treating MPS II for over a decade in Turkey and many other countries globally. Idursulfase has positive effects in prolonging survival and improving or stabilizing somatic manifestations. Although hematopoietic stem cell transplantation (HSCT) has been explored as a potential treatment for MPS II, there remains a substantial lack of comprehensive data regarding its long-term efficacy and outcomes. The existing evidence is limited, which complicates the ability to draw definitive conclusions about the sustained benefits, risks, or overall impacts of HSCT on disease progression, neurological involvement, and the quality of life for patients with MPS II over extended periods [3,4,5].

Newborn screening for MPS II is currently ongoing in Taiwan and has recently been accepted nationwide in the USA [6,7]; however, it is still not included in Turkey’s newborn screening program.

The goal of our report was to present the phenotypic and genotypic characteristics and describe the clinical outcomes of patients with MPS II, a rare condition, after starting ERT at various ages. The patients formed a comprehensive study group from two different regions in Turkey.

## 2. Materials and Methods

### 2.1. Study Design and Subjects

This study involved a cohort of forty-six MPS II patients, recruited from two distinguished centers in Turkey: Ege University Medical Faculty in Izmir and Cukurova University Medical Faculty in Adana. We conducted retrospective data collection from the patients’ medical records, ensuring that all procedures were carried out in line with ethical standards and following approval from the Medical Research Ethics Committee of Çukurova University Faculty of Medicine (document number: 16.09.2022-125-10). Written informed consent to be included in the study was obtained from the participants or the parents of participants under 18. The study followed the principles outlined in the Helsinki Declaration (1964). The data were gathered between January 2022 and December 2023.

The diagnosis of MPS II was confirmed using I2S enzyme analysis and/or *IDS* gene analysis. The clinical data that were collected encompassed a comprehensive range of assessments aimed at evaluating the patients’ overall health and development. This included examining the physical appearance, gathering anthropometric measurements, and conducting detailed neurological and psychological/mental evaluations (Ankara Developmental Screening Test II [up to six years old] or Wechsler Intelligence Scale for Children Revised [WISC-R, 6–16 years]). We also conducted thorough cardiovascular evaluations and pulmonary function tests, alongside eye, ear, nose, and throat (ENT) examinations. The 6-min walk test assessed durability, tailored to the age of the participants, alongside evaluations of the joint range of motion and skeletal involvement through X-ray investigations. All the patients and/or their guardians gave their informed consent for the release of this clinical information.

### 2.2. Urine GAG Analysis

The total urinary GAG levels in spot urine samples were determined spectrophotometrically based on the reaction of urinary GAG with 1,9-dimethyl methylene blue. The GAG levels were corrected with the creatinine in urine [8]. The age-specific normal ranges for the urinary GAG-to-creatinine ratio are as follows: <240 mg/g creatinine for 0–2 months, <200 mg/g creatinine for 2–6 months, <180 mg/g creatinine for 6 months to 1 year, <130 mg/g creatinine for 1–2 years, <110 mg/g creatinine for 2–8 years, <60 mg/g creatinine for 8–12 years, and <30 mg/g creatinine for individuals older than 12 years.

### 2.3. I2S Enzyme Analysis

The enzyme activity of I2S in dried blood spots or plasma was determined using a fluorogenic substrate, 4-methylumbelliferyl-alpha-iduronate-2-sulphate. The enzyme activity was calculated from a calibration curve with 4-methylumbelliferone and expressed in nmol/mL/h [9]. The population-based reference interval for the I2S enzyme is 5–24 nmol/mL/h in dried blood spots and 494–1113 nmol/mL/4 h in plasma.

### 2.4. Molecular Genetic Analysis

Two milliliters of peripheral blood sample was collected in EDTA tubes and stored at −20 °C. Genomic DNA was then isolated from the peripheral blood using standard techniques (QIAamp DNA Blood Mini Kit (Qiagen, Hilden, Germany)). All coding *IDS* (NM_000202.8) exons and their flanking regions were amplified by PCR. Cycle sequencing was performed in the forward and reverse directions with the ABI PRISM Big Dye Terminator Cycle Sequencing Kit (Applied Biosystems, Warrington, UK), following the manufacturer’s instructions, and the sequences were analyzed on the ABI PRISM 3500 DNA Analyzer. The sequencing results were analyzed using the CLC genomic workbench software (version 3.6.5). We analyzed the variants that met our selection criteria in the control group and reserved them for further investigation. Long-range PCR was used to check for rearrangements. Two separate PCRs (long-range PCR) were performed using gel electrophoresis to demonstrate the recombination between the *IDS* gene and its pseudogene (*IDS*2). These PCR reactions were applied to the parents of the affected individuals for a segregation analysis, as well as to two healthy female and two healthy male control subjects. In the first PCR reaction, a 3795-base-pair (bp) PCR product was expected by amplifying exons 7 and 8 of the *IDS* gene. The second PCR reaction aimed to detect a PCR product indicative of possible recombination between the *IDS* gene and its pseudogene. We interpreted the novel variants by meticulously following the guidelines established by the American College of Medical Genetics and Genomics (ACMG). We utilized the Varsome and Franklin platforms to classify these variants accurately (Franklin by Genoox. [(accessed on 23 April 2024)]; available online: https://franklin.genoox.com.) [10,11].

### 2.5. Statistical Analysis

The mean, standard deviation, median, minimum, maximum value frequency, and percentage were used as descriptive statistics. The distribution of variables was measured using the Kolmogorov–Simirnov and Shapiro–Wilk tests. Independent sample *t*-tests were used to analyze independent quantitative data with a normal distribution. The Mann–Whitney U test was used in the analysis of quantitative independent data with non-normal distribution. A paired sample t-test and the Wilcoxon test were used in the analysis of dependent quantitative data. A chi-square test was used to compare the qualitative data. The SPSS 28.0 program was used to conduct the analyses.

## 3. Results

### 3.1. Demographics and Patient Characteristics

The study group included 46 male patients from 40 different families (Table 1).

A total of ten patients had a brother diagnosed with MPS II, while two patients had a cousin with MPS II. While the mean age of diagnosis of the first cases in the families was 3.8 ± 2.0, the mean age of diagnosis of the second cases was 1.1 ± 1.2 years (Table 2).

**Figure 1:**
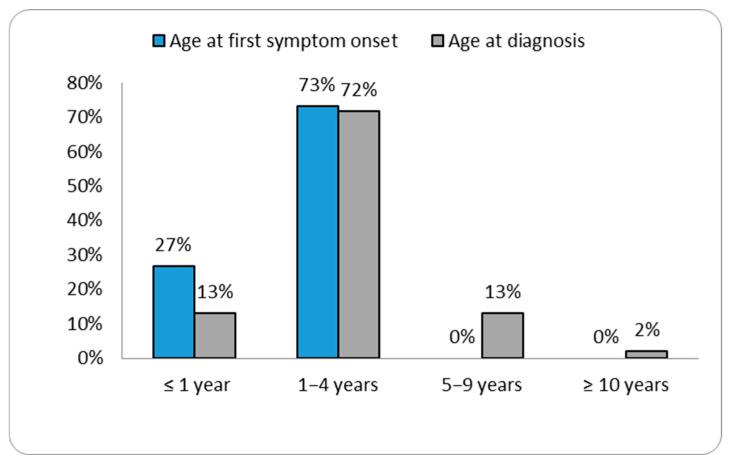
Distribution of cases according to the age at first symptom and age at diagnosis.

**Figure 2:**
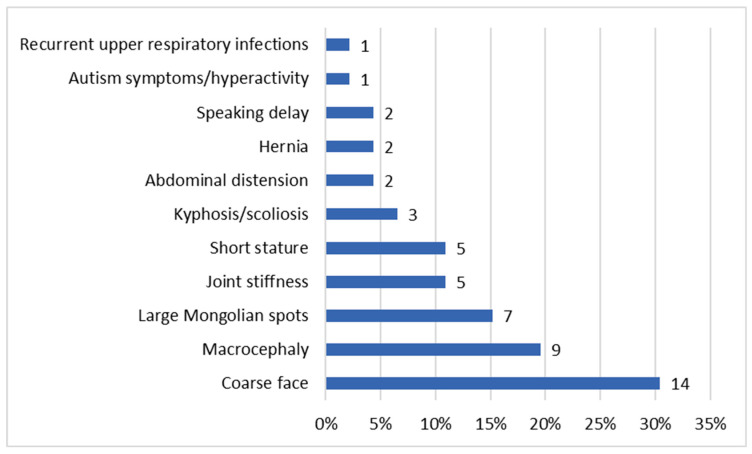
Distribution of first symptoms. The percentages reflect the proportion of patients exhibiting each symptom relative to the total cohort. Since patients may present with multiple symptoms, the totals exceed 100%, as the features are not mutually exclusive.

The ages at diagnosis ranged from two months for patient P46 to 26.5 years for patient P3 (Table 1). Our data showed that the age of diagnosis tended to decrease over the years (Figure 1 and Supplementary Figure S1). Patient P40 was diagnosed with MPS II prior to the emergence of symptoms, as there was a relevant history of the condition in an older sibling already diagnosed with MPS II. In the remaining patient group, the most commonly reported initial signs of MPS II by parents were coarse facial features (30.4%) (see Figure 2).

All patients had enzymatic and/or molecular diagnosis confirmation. The results of the *IDS* gene analysis of 39 patients were obtained from the patient’s medical records. Twenty-five different disease-causing pathogenic variants were identified (Table 3). Twenty patients had missense variants, eight patients had nonsense variants, five had deletions/duplications, four had complex rearrangements, and two had intronic variants.

Notably, in four patients (P6, P20, P27, and P37), no PCR product was obtained in the first reaction, whereas a 2800 bp PCR product was detected in the second reaction, suggesting a recombination event between the *IDS* gene and its pseudogene. In the mothers of these patients, PCR products were obtained in both reactions, confirming their carrier status. In contrast, the fathers and control subjects showed only the expected 3795 bp product in the first reaction, with no product in the second, indicating no recombination.

Among the identified variants, the intronic c.880-8A>G variant was detected in two patients (P9 and P40). This variant is listed as pathogenic in the ClinVar database. Moreover, the in silico splicing prediction tool SpliceAI indicated a strong impact on RNA splicing, potentially resulting in the loss of function or the formation of aberrant transcripts, with a high splice-altering score (1.0). Eight novel pathogenic variants were detected in the *IDS* gene (Table 3).

### 3.2. ERT Journey

Thirty-eight patients were treated with idursulfase (Elaprase^®^, Takeda Pharmaceutical Company Limited, Tokyo, Japan), which was administered intravenously at a 0.5 mg/kg/week dose. Since its introduction in Turkey in 2008, idursulfase infusions have been restricted to secondary or tertiary care hospitals, as home-based administration is not permitted. The median age at the time of starting ERT was 2.8 (0.2–26.6) years. The median duration of treatment with ERT was 6.3 (0.1–13.8) years (Table 1), and 216.5 patient-years of treatment duration was reached. No hypersensitivity reactions or side effects were observed in any patients.

Eight cases in our cohort did not receive ERT. We determined that two patients (P1, P2) were diagnosed and died before the discovery of idursulfase, two patients (P5, P10) did not meet the legal criteria for ERT in Turkey due to severe intellectual disability, and four patients (P32, P38, P41, P45) could not receive treatment because their parents declined ERT.

In the ERT group, organomegaly decreased from 84% (31) to 28% (8) at the last visit. The median urine GAG decreased from 5.2 (min:1–max:67) to 2.2 (min:0.5–max:43.7) as a multiple of the normal for the age. The 6MWT showed an increase from 236 (133–540) meters to 337 (160–700) meters (Supplementary Table S1).

### 3.3. Clinical and Systemic Findings

Seven cases (15.2%) had macrosomia at birth. One patient (P36) showed an increased birth length and head circumference (+2.2 SDS for both). At the time of diagnosis, the mean body weight was 1.0 (±1.5 SD) SDS, the mean height SDS was −0.3 (±1.8 SD), the mean body mass index (BMI) SDS was 1.4 (±1.5 SD), and the mean head circumference SDS was 1.3 (±1.5 SD). The group that did not receive ERT was not included in the analysis because regular monitoring was not continued and the last anthropometric measurement data collected were not sufficient. The patient group who started ERT at ≥3 years of age had lower weight, height, and head circumference SDSs compared to the other group. The last measurement of the height SDS taken showed a significant difference (*p* < 0.05) between the group that started ERT before the age of three and the group that started ERT at the age of three and later (Table 4).

**Table 3:** Disease-causing variants identified in the *IDS* gene.

Number of Patients	DNA Change	Amino Acid Change	Novelty	Pathogenicity	Variant Type	Phenotype(s) in Our Study	Previous Reported Phenotype
4	Complex rearrangement		Known	Pathogenic	Complex rearrangement	Severe	Severe
3	c.262C>T	p.Arg88Cys	Known	Pathogenic	Missense	Severe	Severe
3	*IDS* whole-gene hemizygous deletion		Known	Pathogenic	Gross deletion/duplication	Severe	Severe
2	c.253G>A	p.Ala85Thr	Known	Pathogenic	Missense	Attenuated	Not clear
**2**	**c.880-8A>G**		**Novel**	VUS	Intronic	Severe	No
**2**	**c.254C>A**	**p.Ala85Asp**	**Novel**	Likely pathogenic	Missense	Severe	No
2	c.257C>T	p.Pro86Leu	Known	Pathogenic	Missense	Severe	Severe
2	c.263G>A	p.Arg88His	Known	Pathogenic	Missense	Severe	Severe
2	c.162T>G	p.Tyr54 *	Known	Likely pathogenic	Nonsense	Severe	Severe
2	c.1403G>A	p.Arg468Gln	Known	Pathogenic	Missense	Severe	Severe
**1**	**c.934G>A**	**p.Gly312Ser**	**Novel**	Likely pathogenic	Missense	Severe	No
**1**	**c.362A>C**	**p.Gln121Pro**	**Novel**	Likely pathogenic	Missense	Severe	No
**1**	**c.261C>G**	**p.Ser87Arg**	**Novel**	Likely pathogenic	Missense	Severe	No
**1**	**c.63C>A**	p.Cys21 *	**Novel**	Likely pathogenic	Nonsense	Severe	No
**1**	**c.412C>T**	**p.His138Tyr**	**Novel**	Likely pathogenic	Missense	Attenuated	No
**1**	**c.1010G>A**	p.Trp337 *	**Novel**	Likely pathogenic	Nonsense	Severe	No
1	Exon 9 hemizygous deletion		Known		Gross deletion/duplication	Severe	Severe
1	c.322T>G	p.Tyr108Asp	Known	Likely pathogenic	Missense	Attenuated	Attenuated
1	c.672G>A	p.Gly224Gly	Known	Pathogenic	Missense	Severe	Severe
1	c.928C>T	p.Gln310 *	Known	Likely pathogenic	Nonsense	Severe	Severe
1	c.1327C>T	p.Arg443 *	Known	Pathogenic	Nonsense	Severe	Severe
1	c.187A>G	p.Asn63Asp	Known	Pathogenic	Missense	Attenuated	Attenuated
1	c.22C>T	p.Arg8 *	Known	Pathogenic	Nonsense	Severe	Severe
1	Exon 4-9 deletion				Gross deletion/duplication	Severe	Severe
1	c.514C>T	p.Arg172 *	Known	Pathogenic	Nonsense	Severe	Severe

DNA: deoxyribonucleic acid, VUS: variant of uncertain significance. Previously unreported pathogenic variants are in bold. According to the HGVS (Human Genome Variation Society) recommended mutation nomenclature, the asterisk (*) denotes a stop (termination) codon. As this symbol is part of standard HGVS nomenclature, no additional footnote is required.

**Table 4:** Comparison of anthropometric measurements of two groups whose ERT starting age was under 3 years or 3 years and older.

	ERT												*p*
	Started Before the Age of 3 Years (*n*: 16)						Started at the Age of 3 or After (*n*: 12)						
	Mean ± SD	Median	Min–Max		I.Q–III.Q		Mean ± SD	Median	Min–Max		I.Q–III.Q		
***Weight SDS***													
First visit	1.47 ± 0.85	1.81	−0.66	2.60	0.99	2.17	0.71 ± 2.05	0.83	−3.79	3.50	−0.18	2.20	0.243 ^m^
Last visit	1.51 ± 1.16	1.79	−1.40	3.30	0.66	2.25	−1.98 ± 3.34	−1.58	−11.00	2.60	−2.53	−0.21	***0.001*** **^m^**
First/last variance	0.02 ± 0.89	0.00	−1.80	1.20	−0.50	0.67	−2.62 ± 3.00	−2.13	−9.90	1.07	−4.04	−0.49	***0.005*** **^m^**
*Alteration within the group p*	0.875 ^w^						***0.013*** **^w^**						
***Height SDS***													
First visit	0.59 ± 1.29	0.40	−2.40	2.58	−0.23	1.70	−1.07 ± 1.50	−0.70	−3.79	1.30	−2.12	−0.02	***0.007*** **^m^**
Last visit	0.00 ± 2.08	0.42	−6.40	1.93	−0.43	1.30	−3.70 ± 3.23	−4.06	−11.00	0.70	−5.34	−0.66	***0.004*** **^m^**
First/last variance	−0.54 ± 1.71	0.00	−4.00	1.39	−1.69	0.48	−2.53 ± 2.72	−1.97	−8.62	1.15	−4.39	−0.60	***0.049*** **^m^**
*Alteration within the group p*	0.534 ^w^						***0.010*** **^w^**						
***BMI SDS***													
First visit	1.47 ± 1.03	1.58	−0.86	3.20	1.18	2.10	1.91 ± 1.53	2.60	−0.86	3.69	0.50	3.35	0.669 ^m^
Last visit	1.91 ± 1.24	2.20	−0.86	3.24	1.58	3.04	0.41 ± 1.25	0.50	−2.00	2.90	−0.55	1.16	***0.014*** **^m^**
First/last variance	0.40 ± 0.69	0.40	−0.80	1.67	−0.06	0.96	−1.44 ± 1.13	−1.67	−3.07	0.14	−2.31	−0.09	***0.000*** **^m^**
*Alteration within the group p*	***0.00*** **^w^**						***0.002*** **^w^**						
***Head Circumference SDS***													
First visit	1.39 ± 1.24	1.30	−0.60	3.49	0.49	2.45	0.06 ± 1.53	0.09	−1.79	1.88	−1.57	1.67	0.130 ^m^
Last visit	2.72 ± 1.60	2.68	−0.20	5.11	1.70	3.83	−0.33 ± 0.96	−0.05	−1.79	0.85	−1.30	0.38	***0.002*** **^m^**
First/last variance	0.90 ± 1.07	0.54	0.00	2.70	0.00	2.03	−0.42 ± 1.64	0.00	−3.01	1.42	−1.88	0.83	0.105 ^m^
*Alteration within the group p*	***0.050*** **^w^**						0.598 ^w^						

BMI: body mass index, ERT: enzyme replacement therapy, SD: standard deviation, SDS: standard deviation score. ^m^: Mann–Whitney U test, ^w^: Wilcoxon test. Bold and italic formatting indicate statistically significant *p*-values (*p* < 0.05).

At the baseline visit, 63% (27) of the cases had valve insufficiency and 15% (6) had hypertrophic cardiomyopathy (HCMP). Mitral valve insufficiency was detected in 23 cases, aortic valve insufficiency was detected in 13, tricuspid valve insufficiency was detected in 7, and pulmonary valve insufficiency was detected in 1. The first and last echocardiograms of the cases receiving ERT were compared regarding valve insufficiency. The same was observed in fourteen cases, deterioration was observed in nine cases (new valve involvement or an increase in the degree of existing insufficiency), and a regression in valve insufficiency was observed in two cases. The last echocardiography data collected could only be accessed for two cases that did not receive ERT for a comparison to the first echocardiography. It was observed that valve involvement remained stable (P38) in one patient, while it deteriorated in the other (P5). Five of the six patients with HCMP received ERT, and HCMP regressed in three patients during follow-up.

Upper airway obstruction-related symptoms were present in 49% (22) of the study cohort; 28% (9) had physical examination findings regarding this issue at their baseline visit. During the follow-up period, three cases needed CPAP/BPAP, and the other four cases had to have a tracheostomy. All the patients with a tracheostomy were in the ERT-receiving subgroup. The ages of these cases at the time of the tracheostomy and the corresponding duration of ERT were 9.9/4.4 years, 13.3/5.3 years, 11.8/7.3 years, and 12.3/8.9 years, respectively.

Patients were classified as having the neuropathic form if they exhibited a developmental delay and neurocognitive regression, behavioral changes and sleep disturbances, or epilepsy. Our study cohort included 41 neuronopathic and 5 non-neuronopathic cases (Supplementary Tables S2 and S3). We detected behavioral and neurocognitive problems in 23 patients at diagnosis. Carpal tunnel syndrome was detected in seven patients using electromyography, and the parents of four patients described convulsions at diagnosis.

The results of the cranial MRI are shown in Supplementary Table S4. White matter abnormalities, enlarged Virchow–Robin spaces, and atrophy were the three most common findings at the diagnosis and last evaluation. Seven patients underwent shunting due to hydrocephaly, and one patient underwent spinal cord decompression surgery.

Hearing loss was detected in 70% of the patients (*n* = 32), mostly of a mixed type. In the subgroup that received ERT, 66% had hearing loss at baseline and hearing loss was detected in 57% (16) at the last visit. All of these cases involved using hearing aids, and 38% (12) of them were observed to have ventilation tubes. In the subgroup that did not receive ERT, the frequency of hearing loss was observed to be 88% at baseline and 60% at the last visit. Only one case used a hearing aid.

Thirty cases had adenoid and/or tonsillar hypertrophy in the study group. In the subgroup that received ERT, 70% had it at baseline, and it was detected in 67% of the patients at the last visit. A total of 38% (12) of them were observed to have received an adenoidectomy and/or tonsillectomy. In the subgroup that did not receive ERT, the adenoid and/or tonsillar hypertrophy frequency was 50% at baseline and 60% at the last visit.

Corneal clouding was detected in 7% (3) of the patients (P16, P31, and P35) at baseline visits, and optic disc pathologies were detected in 4% (2) of the patients (H15 and H21). In the cases that received ERT, it was determined that corneal clouding disappeared in one case (P35) at the 46th month of ERT and continued to decrease in one case (P16) at the 125th month of ERT. In two cases with optic disc pathology, it was observed that the optic disc pathology continued in the 103rd and 137th months of ERT.

A total of 62% (26) of the study group had a hernia at the first examination; 88% (23) of them were umbilical, 42% (11) of them were inguinal, and 4% (1) of them were scrotal hernias. Fifteen of these, along with two cases whose hernia developed during follow-up (a total of seventeen cases of the cohort), underwent surgical intervention. At the last visit, a hernia was detected in 45% (17) of the cases in the study group. Despite the surgical correction, it was observed that the hernias recurred in four cases, and all of them were umbilical hernias.

Dysostosis multiplex was observed in 94% (33/35) of the cases using the available imaging data, and restricted joint range of motion was noted in 56% (26/46) of our patients at the time of diagnosis. Thirty cases received physical therapy to preserve and improve their physical function. None of them underwent a surgical approach for the management of their joint involvement.

### 3.4. Survival

**Figure 3:**
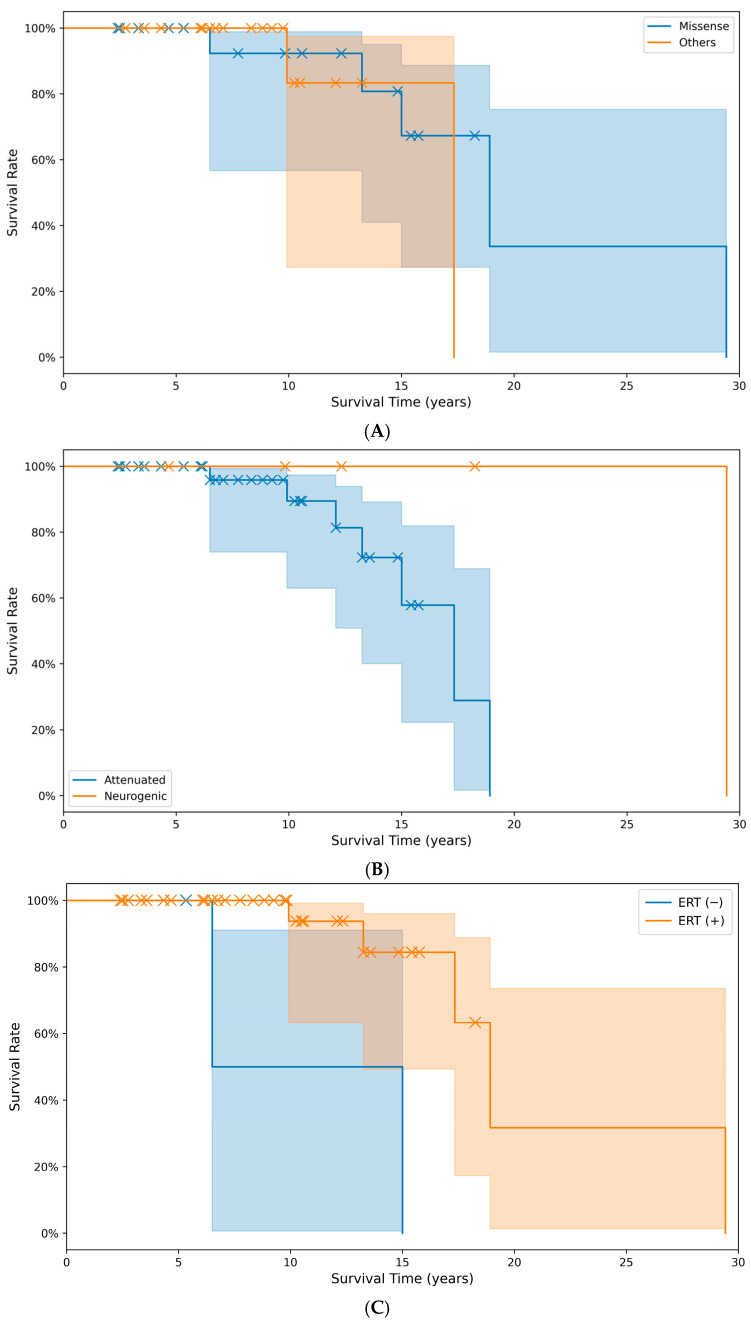
Comparison of cumulative survival rates and survival times across different groups: (**A**) between pathogenic variant types (*p* = 0.059), (**B**) between phenotypes (*p* = 0.079), and (**C**) between treatment groups with or without ERT (*p* = 0.004). X markers indicate events. (Note: A survival event occurred at month 353, beyond the main data range visualized here in (**C**)).

The cumulative survival time did not differ significantly (*p* > 0.05) between the group with missense pathogenic variants (20.3 years) and the group with other pathogenic variant types (16.1 years). The predicted survival time in the non-treatment with ERT (10.8 years) group was significantly (*p* < 0.05) lower than the treatment group with ERT (20.8 years) (Figure 3).

## 4. Discussion

Our study provides a comprehensive overview of the clinical spectrum, genetic diversity, and treatment outcomes of MPS II in a large cohort from Turkey.

An early diagnosis, supported by family history and an increased awareness of symptoms, significantly lowers the age at which subsequent cases within the same family are identified. In our cohort, the average age at diagnosis for the first identified cases was 45 months, whereas the second cases were diagnosed much earlier, at a mean of 13 months. This likely reflects a heightened awareness of MPS II among clinicians and families [12], which is crucial for optimizing the treatment outcomes [12,13,14].

Idursulfase was approved by the FDA in 2006 and by EMA in 2007. It has been available since 2008 and was licensed in our country in 2018. The absence of hypersensitivity reactions suggests good treatment tolerance. However, our study also highlights the difficulties in accessing ERT, with legal and social barriers preventing treatment in some cases, as the HOS reported in 2017 [15]. However, a limitation of our study is the absence of data on the anti-drug antibody (ADA) levels in our patient cohort. The development of ADAs can potentially influence the efficacy and safety of enzyme replacement therapy by reducing drug bioavailability or triggering immune reactions. Although no hypersensitivity reactions were observed in our patients, ADA monitoring could have provided further insight into the treatment response variability. Future studies should consider incorporating ADA assessments to better understand their impact on the long-term treatment outcomes in MPS II patients [16,17].

Our ERT-receiving patients showed improvements in their walking capacity, hepatosplenomegaly, and urinary GAG levels, as reported in the literature [18,19,20]. Managing the neurological involvement remains challenging, as intravenous idursulfase administration was ineffective at treating central nervous system pathology due to the blood–brain barrier [21,22,23]. However, animal models and case reports suggest neurocognitive and neurobehavioral benefits of ERT, including decreased brain GAG accumulation, ameliorated brain tissue damage, and improved behavior in individuals with neuronopathic MPS II. These benefits were associated with earlier initiation and higher doses [14,24,25].

The anthropometric measurements revealed abnormalities in our study group. The patient group who started ERT at ≥3 years of age had lower weight, height, and head circumference SDSs compared to the other group. The last measurement of the height SDS showed a significant difference (*p* < 0.05) between the groups that started ERT before the age of three and those who started ERT at the age of three or later. We also believe that, in MPS II, the overgrowth typically seen in the early years of life tends to diminish with age, and that the progressive neurological decline associated with increasing age negatively impacts nutrition, playing a role in this process [17]. However, our study cohort showed marked growth retardation as they aged, as seen by the height SDS of both the ERT (+) and ERT (−groups. We also saw that ERT had a more positive effect on the height SD when started before age three. According to data from the HOS, MPS II patients who initiated ERT before the age of 18 months demonstrated more favorable outcomes across all clinical parameters [26]. Based on this information and our results, we emphasize the necessity of an early diagnosis and the timely initiation of treatment.

Upper airway obstruction-related symptoms were identified in 49% (22) of the study cohort, and 15.2% (7) required respiratory support interventions, including CPAP, BPAP, or a tracheostomy. All the patients who underwent a tracheostomy belonged to the ERT-treated subgroup. The need for interventions, such as CPAP or BPAP and a tracheostomy, indicates the progressive nature of the respiratory involvement in MPS II [18,19].

Our study cohort consisted of a more severe phenotype group, with cognitive impairment in 89.1%, in contrast with the groups present in the existing literature. The Hunter Outcome Survey (HOS) data show that 55.9% of patients had neurological impairment at any age [20,27].

In our study, *IDS* gene sequencing data were available for 39 patients. A total of 25 different variant types were detected in the study group, and eight were novel. The most common variant identified was a complex rearrangement, constituting 10.2% (4/39) of all variants. This was also the most frequent variant of the *IDS* gene in Mexican, Spanish, Portuguese, and Latin American patients, comprising more than 30 patients [21,22]. However, gross *IDS* rearrangements are associated with more severe MPS II phenotypes, including significant central nervous system impairments [21,23,24,25]. Since the evaluation of the *IDS* gene with standard methods does not show complex rearrangements, a complex rearrangement should be ruled out by using another assay in patients who do not have a disease that causes a pathogenic variant in the *IDS* gene, but who have enzymatic and clinical data compatible with MPS II [22]. Therefore, we suggest that the actual frequency of these complex rearrangements may be underestimated. We used gel electrophoresis to demonstrate the recombination between the *IDS* gene and its pseudogene.

The genotype–phenotype correlation has been reported for some pathogenic variants in the *IDS* gene [28,29,30]. Previous studies have reported that deletion, recombination, frameshift, and, in most cases, nonsense variants were associated with severe MPS II [21,22,23,31,32,33,34,35,36,37,38,39,40,41,42,43,44]. Our results are consistent with these reports. In addition, four missense variants were responsible for the attenuated phenotype, while nine caused the severe phenotype. This finding was the opposite of the results obtained by Seo et al. (7) and Kosuga et al. (49). Furthermore, while the c.253G>A variant has been related to both severe [45] and attenuated [33,45,46] phenotypes in previous studies, we observed an attenuated phenotype in our two cases with this variant. Notably, the level of enzyme activity is not a reliable predictor of the phenotypic severity [35]. Therefore, the debate about missense pathogenic variants will continue.

Discovering eight novel pathogenic variants in the *IDS* gene in our cohort significantly enhances our understanding of MPS II’s genotypic spectrum, providing crucial insights into the disease’s underlying molecular mechanisms.

The survival analysis revealed a significant improvement in the life expectancy with the use of ERT in our study. Bruton et al. reported from the HOS database that the risk of death decreased by 54% in patients who received ERT [47]. Our findings align with this and highlight the critical role of timely and sustained ERT. However, the lack of a significant difference in the survival between patients with missense pathogenic variants and those with other pathogenic variant types, and between severe and mild phenotypes, suggests that factors beyond the genotype, such as access to care and early intervention, may play a pivotal role in the outcomes [46].

## 5. Conclusions

In summary, this study emphasizes the heterogeneity of MPS II and the critical importance of early diagnosis and treatment. Continued research and collaboration are essential to address the challenges in diagnoses, treatment access, and the management of complications in MPS II, ultimately improving the prognosis and quality of life for affected individuals.

## Figures and Tables

**Table 1:** Patient demographics and baseline characteristics.

	Mean ± SD/*n* (%)	Min–Max	Median
**Gender**			
Male	46 (100%)		
Female	0 (0%)		
**Another family member with MPS II**	15 (32.6%)		
**Age at first symptoms (y)**	1.8± 1.1	0.0–4.0	1.8
**Duration between first symptom and diagnosis (y)**	1.6 ± 3.4	0.0–22.5	0.8
**Age at diagnosis (y)**	3.3 ± 3.9	0.2–26.5	2.5
**Age at initiation of ERT (y)**	3.9 ± 4.2	0.2–26.6	2.8
**Duration of ERT (y)**	6.2 ± 3.7	0.1–13.8	6.1
**Current age (y)**	10.0 ± 5.6	2.4–29.4	9.8
**Subtype**			
Severe	41 (89.1%)		
Attenuated	5 (10.9%)		
**ERT**			
(+)	38 (82.7%)		
(−)	8 (17.3%)		

ERT: enzyme replacement therapy, max: maximum, min: minimum, SD: standard deviation, y: years.

**Table 2:** Phenotypic presentation and clinical features identified through family screening, with clinical findings observed after ERT.

			1. Family		2. Family		3. Family		4. Family		5. Family		6. Family	
			1. Index (P9)	1. FS (P40)	2. Index (P10)	2. FS (P13)	3. Index (P20)	3. FS (P27)	4. Index (P21)	4. FS (P43)	5. Index (P30)	5. FS (P36)	6. Index (P32)	6. FS (P41)
At diagnosis	Age (y)		5.8	0.2	5.7	3.0	5.2	2.3	2.4	0.3	2.0	0.8	1.5	0.3
	Systematic	Height (SDS)	−3.0	1.12	−4.9	−1.50	0.79	−0.59	−2.40	0.62	−1.80	2.58	N.A	1.30
		Cardiac	MI, TI	Normal	AI, MI	MI	AI, MI	AI, TI, PI	AI, MI	Normal	AI, MI	Normal	Normal	Normal
		Visceromegaly	Yes	Yes	Yes	Yes	Yes	Yes	Yes	No	Yes	Yes	Yes	No
		Neurological	BP, CP, E	Normal	BP, CP, AS	Normal	BP, CP, AS	Normal	Normal	Normal	BP, CP	BP	BP, CP, AS	Normal
	GAG fold		1.6	9.7	7.5	1.9	10.6	5.5	4.9	8.1	3.9	6.9	2.6	18.5
ERT	Age (y)		6.1	0.2	No ^§^	4.8	5.5	2.8	2.4	0.3	2.1	1.1	No ^¥^	No ^¥^
	Duration (y)		11.3	4.2	(-)	10.1	6.5	6.5	8.6	3.0	6.3	5.4	(-)	(-)
	Side effects		No	No	No	No	No	No	No	No	No	No	No	No
Findings after ERT	Age (y)		17.3	4.3	6.5	14.8	12.1	9.3	10.6	3.3	8.3	6.5	3.6	2.3
	Systematic	Height (SDS)	−4.0	0.2	N.A	−2.9	−3.6	0.8	−6.4	0.4	0.3	−1.1	0.3	N.A
		Cardiac	MI, TI	AI	N.A	MI	AI, MI, PI	AI	AI, MI	Normal	MY, TY	MY, TY	MY	N.A
		Visceromegaly	Yes	No	N.A	No	Yes	Yes	Yes	No	No	No	Yes	Yes
		Neurological	BP, CP, E	N.A	N.A	BP, CP	BP, CP, AS	Normal	BP, CP, AS	Normal	BP, CP, AS	BP, CP, E	BP, CP, AS	N.A
	GAG fold		0.97	4.8	N.A	0.6	7.5	0.91	2.34	1.89	1.8	1.5	0.70	N.A

AD: abdominal distension, AI: aortic insufficiency, AS: autism symptoms, BP: behavioral problems, CP: cognitive problems, CF: coarse face, E: epilepsy, ERT: enzyme replacement therapy, FS: family screening, H: hernia, LMS: large Mongolian spot, M: macrocephaly, MI: mitral insufficiency, N.A: not available, PI: pulmonary insufficiency, RUAI: recurrent upper respiratory infections, SD: speaking delay, SS: short stature, TI: tricuspid insufficiency, y: years. ^§^: did not meet the legal criteria, ^¥^: parent rejection.

## Data Availability

The datasets generated and analyzed during the current study are available in the Human Gene Pathogenic Variant Database, at www.hgmd.cf.ac.uk/ (accessed on 1 November 2024) (information about nucleotide changes described before), and ClinVar, at https://www.ncbi.nlm.nih.gov/clinvar/ (accessed on 1 November 2024) (information about novel nucleotide changes; accession numbers SCV001450592-SCV001450635) (see also Table 2). The information about *IDS* gene sequencing is available at https://www.ncbi.nlm.nih.gov/gdv/browser/gene/?id=3423 (accessed on 1 November 2024) (NC_000023.11).

## Supplementary Materials

The following supporting information can be downloaded at https://www.mdpi.com/article/10.3390/diagnostics15212773/s1. Table S1. Clinical and biochemical outcomes of ERT in ERT-receiving group. Table S2. Clinical, enzymatic, and molecular features of cases. Table S3. Clinical features of non-neuronopathic group (attenuated). Table S4. Cranial MRI findings of patients with MPS II. Figure S1. Distribution by age at diagnosis by date of birth. * A non-neuronopathic case diagnosed at an adult age was not included. The black line shows the trendline.

## Abbreviations

The following abbreviations are used in this manuscript:

BMI	Body mass index
DNA	Deoxyribonucleic acid
DS	Dermatan sulfate
ERT	Enzyme replacement therapy
GAG	Glycosaminoglycan
HCMP	Hypertrophic cardiomyopathy
HSCT	Hematopoietic stem cell transplantation
HOS	Hunter Outcome Survey
HS	Heparan sulfate
I2S	Iduronate-2-sulfatase
MPS II	Mucopolysaccharidosis type II
MRI	Magnetic resonance imaging
SDS	Standard deviation score
6MWT	6-min walk test
