# Supplementary material for: Genotype–Phenotype Correlations and Shifting Diagnosis Age in Turkish Mucopolysaccharidosis Type II Patients: A Multicenter Retrospective Study
Source: Diagnostics (Basel). 2025 Oct 31;15(21):2773. doi: 10.3390/diagnostics15212773 (PMC12607719; doi:10.3390/diagnostics15212773)
Supplement: Supplementary file 1 [file diagnostics-15-02773-s001.zip › diagnostics-3934192-update-final-supplementary.pdf]

Supplementary Table S1. Clinical and biochemical outcomes of ERT in ERT-receiving group.

Patient number	Age at diagnosis (y)	GAG fold increase at diagnosis	Baseline visit Weight SDS	Baseline visit Height SDS	Baseline visit HC SDS	Age at initiation of ERT (y)	Duration of ERT (y)	6MWT	GAG fold inrease at last visit	Last visit Weight SDS	Last visit Height SDS	Last visit HC SDS
P3	26.5	1.7	-3.79	-3.79	-1.79	26.6	2.8	N.A.	0.8	-4.28	-5.51	-1.79
P4	1.4	4.8	N.A.	N.A.	N.A.	7.9	5.3	wheelchair dependent	2.0	-2.7	-4.7	N.A.
P6	4.8	7.3	1.76	-2.12		5.5	4.4	wheelchair dependent	N.A.	-1.37	-4.09	N.A.
P7	1.9	8.3	-1.10	-2.38	1.88	6.8	12.1	wheelchair dependent	0.8	-11	-11	-1.13
P8	4.0	N.A.	0.52	-0.02	0.43	4.9	13.8	240 meters	N.A	-1.5	-1.79	N.A.
P9λ	5.8	N.A.	-0.55	-3.00	N.A.	6.1	11.3	wheelchair dependent	N.A.	N.A.	N.A.	N.A.
P11	4.3	3.8	1.00	-2.00	-1.50	4.4	11.4	wheelchair dependent	2.1	-1.13	-4.02	-0.08
P12	2.3	N.A.	2.20	1.70	1.30	2.3	13.6	wheelchair dependent	6.3	0.4	N.A.	N.A.
P13α	3.0	1.9	0.09	-1.50	N.A.	4.8	10.1	walking with support	N.A.	N.A.	N.A.	N.A.
P14	3.2	N.A.	0.88	-2.20	-0.63	3.3	N.A.	N.A.	N.A.	N.A.	N.A.	N.A.
P15	2.8	N.A.	1.29	0.28	N.A.	3.0	N.A.	wheelchair dependent	4.3	N.A.	N.A.	N.A.
P16	3.0	1.0	1.75	-0.64	N.A.	3.2	10.4	wheelchair dependent	9.0	N.A.	N.A.	N.A.
P17	3.3	2.6	0.83	-1.77	N.A.	3.3	9.9	wheelchair dependent	13.3	-2	-5.4	N.A.
P18	2.0	5.8	1.30	-1.40	-0.40	2.3	N.A.	N.A.	N.A.	N.A.	N.A.	N.A.
P19	5.7	2.3	-0.07	-0.70	N.A.	5.8	6.6	500 meters		1	0.45	
P20β	5.2	10.6	3.10	0.79	N.A.	5.5	6.5	225	7.5	-1.65	-3.6	0.22
P21ω	2.4	4.9	-0.66	-2.40	0.84	2.4	8.6	N.A.	2.3	-1.4	-6.4	N.A.
P22	4.4	6.2	3.50	1.30	N.A.	4.4	6.3	N.A.	4.9	2.6	0.7	N.A.
P23	5.2	67.3	2.20	-0.47	1.60	5.9	4.6	wheelchair dependent	N.A.	-1.84	-5.14	0.85
P24	1.3	1.1	2.00	0.50	2.80	1.4	8.4	N.A.	N.A.	N.A.	N.A.	N.A.

P25	2.2	2.4	0.99	-0.46	N.A.	2.3	8.1	N.A.	8.1	1.6	-0.43	N.A.
P26	1.2	1.2	1.86	0.40	0.21	1.2	7.7	294 meters	12.6	1.84	-1.95	N.A.
P27β	2.3	5.5	1.10	-0.59	N.A.	2.8	6.5	200 meters	0.9	2.3	0.8	2.77
P28	9.0		-3.40	-4.00	N.A.	9.0	N.A.	N.A.	N.A.	N.A.	N.A.	N.A.
P29	1.7	1.7	1.10	0.24	2.13	2.0	6.1	550 meters	0.5	1	0.42	2.58
P30π	2.0	3.9	2.20	1.80	2.76	2.1	6.3	400 meters	1.8	3.03	0.33	5.11
P31	1.8	3.7	1.30	-0.20	-0.60	1.8	5.3	700 meters	2.2	1.74	1	
P33	2.4	7.4	2.10	0.80	2.10	2.6	4.3	380 meters	1.0	3.3	1.8	4.8
P34	1.0	9.4	0.99	1.15	3.20	1.4	N.A.	N.A.	N.A.	N.A.	N.A.	N.A.
P35	2.8	6.7	2.60	0.29	0.89	2.8	3.8	N.A.	3.8	2.1	0.59	
P36π	0.8	6.9	2.17	2.58	2.89	1.1	5.4	N.A.	1.5	0.61	-1.18	3.51
P37	2.7	10.1	1.81	-0.23	0.77	2.8	3.5	252 meters	2.0	2.48	-0.32	1.85
P39	3.3	6.4	-0.18	-0.66	-0.26	3.3	1.9	500 meters	0.8	0.1	-0.28	-0.02
P40λ	0.2	N.A	0.28	1.12	2.00	0.2	4.2	N.A.	4.8	N.A.	N.A.	N.A.
P42	2.3	N.A	0.82	1.93	-0.20	2.3	1.8	N.A.	2.2	0.82	1.93	-0.2
P43ω	0.3	8.1	0.04	0.62	2.50	0.3	3.0	N.A.	2.2	N.A.	N.A.	N.A.
P46	2.5	7.7	0.55	1.68	1.50	2.5	0.8	N.A.	N.A	0.55	1.68	1.5

N.A.: Data not available. Patients sharing the same symbol (e.g., β, π, λ, ω) belong to the same family.

Supplementary Table S2. Clinical, enzymatic and molecular features of cases.

**Patient number**	**Consanguinity of parents**	**Family history**	**First symptom(s)**	**Age of first symptoms (y)**	**Diagnosis age (y)**	**Age at initiation of ERT (y)**	**Duration of ERT (y)**	**Current age (y)**	**Disease subtype**	**I2S enzyme level (sample type)**	**Nucleotide variant found in *IDS* gene**
P1	No	No	Coarse face	1.0	1.0	(-)	(-)	12.1, deceased	Severe	0 nmol/ml/4h (P)	Not available
P2	No	No	Speaking delay	2.0	4.4	(-)	(-)	Unknown	Severe	0 nmol/ml/4h (P)	Not available
P3	Yes	No	Short stature	4.0	26.5	26.6	2.8	29.4, deceased	Attenuated	0.37 nmol/ml/4h (P)	c.322T>G
P4	No	No	Coarse face	1.0	1.4	7.9	5.3	13.2, deceased	Severe	0.5 nmol/4h/mg protein (P)	c.262C>T
P5	No	No	RUAI	0.3	2.8	(-)	(-)	15, deceased	Severe	0 nmol/mg/ml (P)	c.262C>T
P6	No	No	Coarse face, speaking delay	2.0	4.8	5.5	4.4	9.9, deceased	Severe	0.62nmol/4h/mg protein (P)	complex rearrangement

P7	No	No	Abdominal distension	0.5	1.9	6.8	12.1	18.9, deceased	Severe	0 nmol/4h/mg protein (F)	c.262C>T
P8	Yes	No	RUAI	3.0	4.0	4.9	13.8	18.3	Attenuated	Not available	c.253G>A
P9	Yes	Brother	Coarse face, hernia	3.0	5.8	6.1	9.6	17.3, deceased	Severe	0 nmol/ml/4h (P)	c.880-8A>G
P10	No	Brother	Speaking delay	2.0	5.7	(-)	(-)	6.5, deceased	Severe	0 nmol/ml/4h (P)	c.257C>T
P11	Yes	No	Speaking delay	3.0	4.3	4.4	11.4	15.8	Severe	0.06 nmol/4h/mg protein (P)	c.672G>A
P12	No	No	Coarse face, hernia	0.7	2.3	2.3	13.6	15.4	Severe	0.59 nmol/4h/mg protein (P)	c.261C>G
P13	No	Brother	Coarse face	2.0	3.0	4.8	10.1	14.8	Severe	0 nmol/ml/4h (P)	c.257C>T
P14	No	No	Coarse face	2.0	3.2	3.3	Unknown	Unknown, deceased	Severe	8.8 nmol/ml/4h (P)	Not available
P15	No	No	Speech and walking disability	2.0	2.8	3.0	Unknown	Unknown	Severe	3.7 nmol/ml/4h (P)	Not available
P16	No	No	Abdominal distension	1.7	3.0	3.2	10.4	13.6	Severe	18.1 nmol/ml/4h (P)	Not available
P17	No	No	RUAI	2.8	3.3	3.3	9.9	13.2	Severe	0 nmol/ml/4h (P)	c.63C>A
P18	No	No	Abdominal distension	1.4	2.0	2.3	Unknown	Unknown	Severe	0.26 nmol/ml/4h (P)	Not available
P19	No	No	Joint stiffness	4.0	5.7	5.8	6.6	12.3	Attenuated	0 nmol/ml/4h (P)	c.187A>G
P20	No	Brother	Speaking delay	3.0	5.2	5.5	6.5	12.1	Severe	0 nmol/ml/4h (P)	complex rearrangement
P21	No	Brother	Abdominal distension	2.0	2.4	2.4	8.6	10.6	Severe	0 nmol/ml/4h (P)	c.1403 G>A
P22	No	No	Hyperactivity	3.0	4.4	4.4	6.3	10.3	Severe	1 nmol/ml/4h (P)	IDS whole gene hemizygous deletion
P23	No	No	Speaking delay	3.0	5.2	5.9	4.6	10.5	Severe	0 nmol/ml/4h (P)	IDS whole gene hemizygous deletion
P24	No	No	Macrocephaly	1.2	1.3	1.4	8.4	9.8	Attenuated	0 nmol/ml/4h (P)	c.412C>T
P25	No	No	Coarse face, joint stiffness	1.7	2.2	2.3	8.1	9.8	Severe	0.01 nmol/mg/ml (P)	c.1010G>A
P26	No	No	RUAI	0.8	1.2	1.2	7.7	8.8	Severe	0 nmol/ml/4h (P)	exon 4-9 deletion
P27	No	Brother	Coarse face	Unknown	2.3	2.8	6.5	9.3	Severe	0 nmol/ml/4h (P)	complex rearrangement

P28	Yes	No	Macrocephaly	3.0	9.0	9.0	Unknown	Unknown, deceased	Severe	4.5 nmol/ml/4h (P)	Not available
P29	No	No	Abdominal distension	0.5	1.7	2.0	6.1	7.8	Severe	0.71 nmol/4h/mg protein (P)	c.263G>A
P30	Yes	Other	RUAI	0.5	2.0	2.1	6.3	8.3	Severe	0.3 nmol/mg/h (P)	c.162T>G
P31	Yes	No	Large mongolian spot, coarse face	1.5	1.8	1.8	5.3	7.1	Severe	0 nmol/ml/4h (P)	c.514C>T
P32	Yes	Brother	Large mongolian spot, kyphoscoliosis	0.1	1.5	(-)	(-)	3.6	Severe	0 nmol/ml/4h (P)	c.254C>A
P33	No	No	Abdominal distension	2.4	2.4	2.6	4.3	6.8	Severe	0.16 nmol/mL/h (DBS)	c.928C>T
P34	Yes	No	Coarse face	0.3	1.0	1.4	Unknown	Unknown	Severe	0 nmol/ml/4h (P)	IDS whole gene hemizygous deletion
P35	Yes	No	Abdominal distension	1.2	2.8	2.8	3.8	6.2	Severe	0.1 nmol/ml/4h (P)	exon 9 hemizygous deletion
P36	No	Other	Macrocephaly	0.0	0.8	1.1	5.4	6.5	Severe	0.12 nmol/mL/h (DBS)	c.162T>G
P37	No	No	Speaking delay	2.0	2.7	2.8	3.5	6.1	Severe	0.04 nmol/mL/h (DBS)	complex rearrangement
P38	No	No	Autism symptoms	2.0	3.4	(-)	(-)	5.3	Severe	0.12 nmol/mL/h (DBS)	c.934G>A
P39	Yes	Brother	Abdominal distension	2.5	3.3	3.3	1.9	4.7	Attenuated	Not available	c.253G>A
P40	Yes	Brother	No symptom	(-)	0.2	0.2	4.2	4.3	Severe	Not available	c.880-8A>G
P41	Yes	Brother	Inguinal hernia	0.0	0.3	(-)	(-)	2.5	Severe	0.05 nmol/mL/h (DBS)	c.254C>A
P42	No	No	Coarse face	1.5	2.3	2.3	1.8	3.6	Severe	Not available	c.1327C>T
P43	No	Brother	Macrocephaly	0.0	0.3	0.3	3.0	3.3	Severe	Not available	c.1403G>A
P44	No	No	Coarse face	2.0	2.5	2.5	0.8	2.8	Severe	0 nmol/ml/4h (P)	c.22C>T
P45	No	No	Coarse face	1.6	1.6	1.8	0.1	2.5	Severe	0 nmol/ml/h (P)	c.263G>A
P46	No	No	Macrocephaly	1.6	2.3	2.3	1	2.4	Severe	0 nmol/ml/4h (P)	c.362A>C

DBS: dried bloodspot, F: fibroblast, M: male, P: plasma, RUAI: recurrent upper airway infections, y: years.

Supplementary Table S3. Clinical features of non-neuronopatic group (atenue).

Patient	First Symptom	Age of first symptom (y)	Age of diagnosis (y)	Time from first symptom to diagnosis (y)	Current age (y)
P3	Recurrent UAI/LAI	4.0	26.5	22.5	29.4 (ex)
P8	Coarse face	3.0	4.0	1.0	18.3
P19	Hernia	4.0	5.7	1.7	12.3
P24	Macrosephaly	1.2	1.3	0.2	9.8
P39	Hepatosplenomegaly	2.5	3.3	0.8	4.7
**Median (min-max)**		**3.0 (1.2-4.0)**	**4.0 (1.3-26.5)**	**1.0 (0.2-22.5)**	**12.3 (4.7-29.4)**

LAI: lower airway infections; UAI: upper airway infections.

Supplementary Table S4. Cranial MRI findings of patients with MPS II.

MRI finding	Diagnosis time (N = 21)		Last visit (N = 26)	
	n	%	n	%
White matter abnormalities	12	57.1%	19	73.1%
Enlarged perivascular Virchow-Robin spaces	8	38.1%	12	46.2%
Atrophy	7	33.3%	16	61.5%
Enlarged CSF spaces	2	9.5%	7	26.9%
Enlarged III ventricle	3	14.3%	13	50.0%
Enlarged lateral ventricles	4	19.0%	12	46.2%
Hydrocephalus	2	9.5%	7	26.9%
J-shaped sella turcica	1	4.8%	1	3.8%
Hyperosteosis of the calvarium	3	14.3%	6	23.1%
Craniocervical stenosis	1	4.8%	6	23.1%
Spinal cord compression	1	4.8%	5	19.2%

Percentages were calculated based on the total number of patients who underwent MRI at each time point.

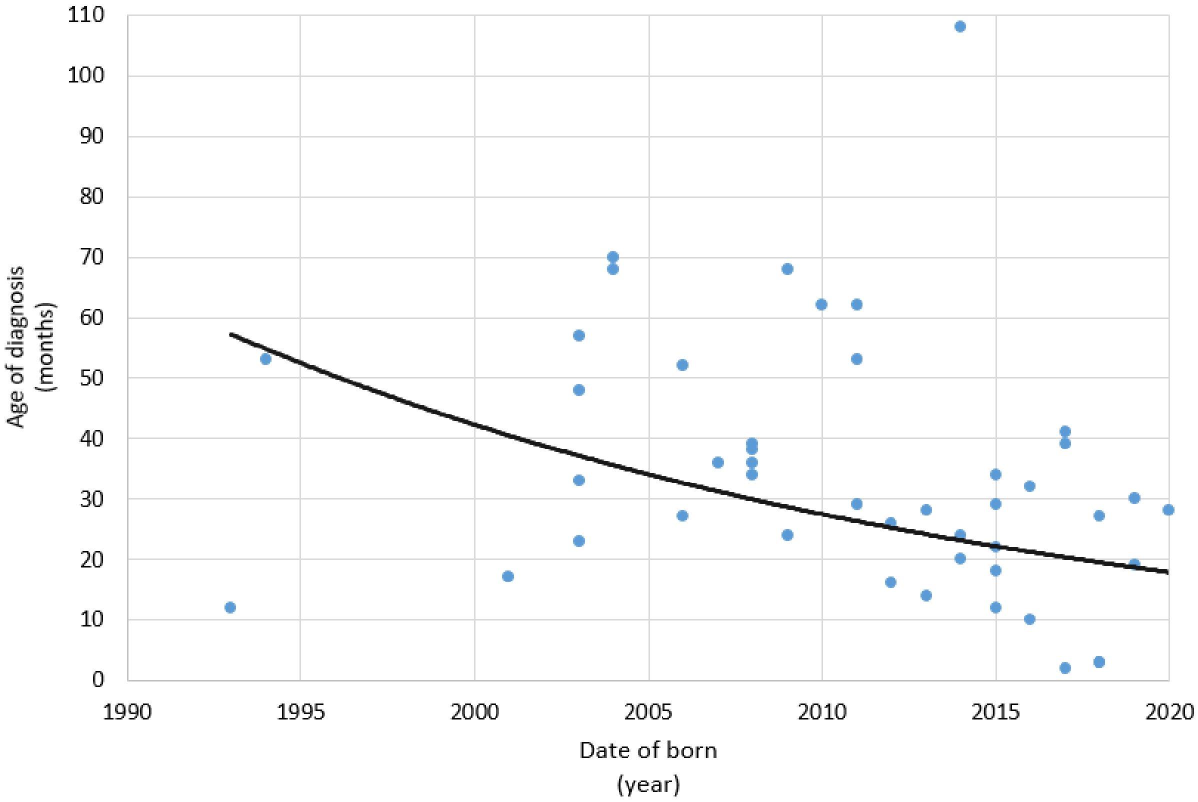

Supplementary Figure S1. Age at diagnosis distribution by date of birth.

*A non-neuronopathic form case diagnosed at an adult age was not included. Black line shows the trendline.
